# Identification and Characterization of a Thermotolerant TILLING Allele of Heat Shock Binding Protein 1 in Tomato

**DOI:** 10.3390/genes10070516

**Published:** 2019-07-07

**Authors:** Dominik Marko, Asmaa El-shershaby, Filomena Carriero, Stephan Summerer, Angelo Petrozza, Rina Iannacone, Enrico Schleiff, Sotirios Fragkostefanakis

**Affiliations:** 1 Department of Biosciences, Molecular Cell Biology of Plants, Goethe University, D-60438 Frankfurt am Main, Germany; 2 ALSIA Research Center Metapontum Agrobios S.S. Jonica 106 Km 448,2 -75100 Matera, Italy; 3 Department of Molecular Biology, Genetic Engineering and Biotechnology Division, National Research Centre, 12311 Dokki, Giza, Egypt; 4 Frankfurt Institute of Advanced Studies (FIAS), D-60438 Frankfurt am Main, Germany; 5 Buchmann Institute for Molecular Life Sciences (BMLS), Goethe University, D-60438 Frankfurt am Main, Germany

**Keywords:** *Solanum lycopersicum*, heat stress, thermotolerance, heat shock protein, heat stress transcription factor, phenotyping, HSBP

## Abstract

The identification of heat stress (HS)-resilient germplasm is important to ensure food security under less favorable environmental conditions. For that, germplasm with an altered activity of factors regulating the HS response is an important genetic tool for crop improvement. Heat shock binding protein (HSBP) is one of the main negative regulators of HS response, acting as a repressor of the activity of HS transcription factors. We identified a TILLING allele of *Solanum lycopersicum* (tomato) *HSBP1*. We examined the effects of the mutation on the functionality of the protein in tomato protoplasts, and compared the thermotolerance capacity of lines carrying the wild-type and mutant alleles of *HSBP1*. The methionine-to-isoleucine mutation in the central heptad repeats of HSBP1 leads to a partial loss of protein function, thereby reducing the inhibitory effect on Hsf activity. Mutant seedlings show enhanced basal thermotolerance, while mature plants exhibit increased resilience in repeated HS treatments, as shown by several physiological parameters. Importantly, plants that are homozygous for the wild-type or mutant *HSBP1* alleles showed no significant differences under non-stressed conditions. Altogether, these results indicate that the identified mutant *HSBP1* allele can be used as a genetic tool in breeding, aiming to improve the thermotolerance of tomato varieties.

## 1. Introduction

Heat stress (HS) is one of the most devastating environmental stresses that a plant can face during its life cycle. At the cellular level, HS impacts membrane fluidity, microtubule organization and activity, and the general stability of enzymes participating in a variety of physiological processes [1,2,3]. Consequently, high temperatures negatively affect the growth of vegetative and floral organs, induce flower abortion, and cause deviations from physiological developmental transitions, including gametophytic defects [4,5,6,7]. 

At the molecular level, HS causes the accumulation of misfolded proteins, which is a condition referred to as proteotoxicity. This hampers the functionality and stability of structural, enzymatic, and regulatory proteins [8]. Protection and recovery from HS depend on the activation of a complex network of molecular responses, which are collectively called the HS response (HSR). A major feature of HSR is the transcriptional upregulation of hundreds of genes coding for proteins with a variety of biological functions, including reactive oxygen species scavengers, hormone metabolism, transcription and translation, signaling, and protein fate [9,10,11,12,13,14]. Among them, a special focus has been placed on heat shock proteins (Hsps), acting as molecular chaperones, for their essential role in the maintenance of protein homeostasis under both physiological and stress conditions [15,16,17].

The majority of HS-induced genes are controlled by members of the HS transcription factor (Hsf) family [18,19]. Plants encode for a large number of Hsfs, which, based on the structure, domain, and functional peculiarities are categorized in classes A, B, and C [20,21]. In plants, HsfA1 members are considered as essential regulators of the initial response and basal thermotolerance; these are required for the upregulation of stress-induced Hsfs, which further contribute to stress response maintenance and stimulation [11,22,23,24,25,26]. Class B Hsfs are mostly involved in the repression of HSR during attenuation after heat stress, but in some cases, a co-activator function with class-A Hsfs has been reported as well [27,28,29,30]. The function of the class C Hsfs remains to be explored. 

The misregulation of Hsf and chaperone networks cause deviations from physiological growth and development both in vegetative and reproductive tissues [11,30,31,32]. Therefore, Hsfs are embedded in a regulatory network involving different layers of control mechanisms that adjust their activity based on the cellular demands. Such a post-translational mechanism has been exemplified on the level of interaction of Hsf with chaperones, co-chaperones, and other associated factors that eventually control the activity, stability, and nucleocytoplasmic equilibrium of Hsfs [33,34,35,36,37,38].

Heat shock binding protein (HSBP) is a conserved eukaryotic protein that primarily acts as a negative regulator of Hsfs via interaction with the oligomerization region of Hsfs [39]. The HSBP monomer has an α-helical structure that can form trimers and hexamers via coiled-coil interactions [39,40]. The nuclear translocation of HSBP upon stress and during recovery from HS is related to the inactivation of Hsfs [41]. It has been proposed that HSBP modulates the attenuation phase of HSR. *Arabidopsis thaliana* HSBP, *Zea mays* EMP2/HSBP1, and *Oryza sativa* HSBP1 and HSBP2 are induced by HS, but also show enhanced expression under physiological conditions in siliques, embryos, and panicles, respectively [41,42,43]. Maize EMP2/HSBP1 functions in the early stages of kernel development under physiological conditions, which is consistent with a developmental role [42]. Interestingly, HSBP only interacts with class A Hsfs, and the different HSBPs show a specificity for different HsfA members. Maize EMP1/HSBP1 interacts with HsfA2e, HsfA3, HsfA4d, and HsfA5, while HSBP2 binds to HsfA2c and HsfA4a [43]. Consistent with a distinct interaction profile that pairs OsHSBP1 and OsHSBP2 with different Hsfs, transgenic lines show differences in the transcript regulation of Hsf-dependent genes such as Hsps [44].

We explored the capacity of ethyl methanesulfonate (EMS)-induced mutations in the HSBP coding gene to have a positive impact on the thermotolerance of tomato plants. Tomato is an economically and dietary important crop world-wide, and has long served as a model plant for flesh fruit development [45]. Considering that tomato is cultivated in areas that are and will be heavily affected by global warming (e.g., the Mediterranean basin) the identification or development of heat-resilient germplasm is of utmost importance for farmers and consumers. Through a TILLING screening performed on a Red Setter mutant population, we identified a mutation in the tomato *HSBP1* gene. This mutation causes a Met to Ile substitution in one of the helical heptad repeats. The effect of the mutation on the HSBP activity on important Hsfs was examined in tomato protoplasts, while the impact of the mutation on plant performance under high temperatures was examined by monitoring the physiological parameters of thermotolerance. We observed a reduction of the negative regulation of the HSBP mutant on Hsf activity resulting in plants with increased thermotolerance in the absence of significant phenotype alterations under non-stressed conditions. Thereby, the *HSBP1* mutant line that is identified can be further used for the genetic improvement of thermotolerance in tomato.

## 2. Materials and Methods 

### 2.1. Plant Material and Stress Treatment

Phenotyping was performed in a greenhouse under controlled conditions via a Scanalyzer 3D platform (LemnaTec Gmbh) in 2-L pots, containing 1.5 kg of soil (50:50 peat moss and river sand). Before sowing, 30 units of nitrogen, 40 units of anhydrous phosphate, and 30 units of potassium oxide were added to the substrate mixture. Growth conditions were 25/20 °C day/night temperature, 65%, relative humidity with a 16 h per day photoperiod. Plants were irrigated with 100 ml of water every 3 days during the analyses. For leaf surface temperature monitoring, plants were either kept at 25 °C or exposed to a gradual temperature increase from 8:00 to 13:00, at which point the temperature reached 36 °C, remained there for 1 hour, and then gradually declined to 25 °C until 18:00. The temperature was recorded on either the youngest fully emerged leaf, or the third oldest leaf of each plant using a PAM 2500 (Walz, Germany).

### 2.2. TILLING Screening

For the identification of induced point mutation in *HSBP1*, a TILLING platform based on Red Setter cultivar was used [46]. DNA amplification was performed using nested PCR with gene-specific primers (HSBP1-For-ext: GGCCCTTTAAAGAACTCTCTCTG, HSBP1-Rev-ext: ATAGGCGGGTGTAGGGTTCT, HSBP1-For-int: TTGGTTCAATTTTCATGCACTT, HSBP1-Rev-int: AAAAAGGCTATAAATTTTCTATTATTGC. Internal primers were 5’-end labeled with IRDye 700 and IRDye 800 dye (LI-COR, Lincoln, NE, USA), respectively. The PCR amplifications were carried out according to the experimental conditions described previously [47]. Mutation detection was performed by using Endonuclease ENDO I [48] and LI-COR 4300 DNA Analyzer (LI-COR, Lincoln, NE, USA). Adobe Photoshop software was used for image analysis (Adobe Systems Inc., San Jose, CA, USA). Mutation was validated by Sanger sequencing, and its position was defined at nt 761 from the first nucleotide of the amplicon generated by primers HSBP1-For-ext/HSBP1-Rev-ext. Prediction of the impact of amino acid change on protein function was done using SIFT software [49].

### 2.3. Genotyping of Mutant Plants

The genotyping of plants was performed as previously described [50]. M3 seeds of families containing the *HSBP1* mutant allele (*HSBP1m*) were grown in a greenhouse under standard conditions and confirmed by Sanger sequencing. Homozygous plants for G761A *HSBP1* mutation were identified and backcrossed to the Red Setter parent line. BC1F1 plants were selfed, and BC1F2 progenies were genotyped for G761A *HSBP1* mutation. Using homozygous BC1F2 mutant plants, a further selfing was adopted to obtain BC1F3 seed stocks. BC1F3 progeny carrying the wild-type *HSBP1* allele were used as control plants and are referred to here as *HSBP1wt*.

### 2.4. Seedling Thermotolerance

Four-day-old seedlings were germinated in the dark at 25 °C on wet paper towels in sealed petri dishes, and were exposed to 25 °C, 39 °C, 42 °C, or 45 °C for 1 hour in a water bath. Thermotolerance was evaluated by measuring the hypocotyl length for the following 7 days. 

### 2.5. Image Based Phenotyping: Data Acquisition and Processing

Phenotyping through image analysis was performed with a Scanalyzer 3D System (LemnaTec GmbH). Plants from each genotype were divided into two groups: control (non-stressed) and heat-shocked (1 hour at 39 °C) daily for four days. Visible light images of the plants were captured immediately after heat shock treatment.

The imaging involving three mutually orthogonal vantage points was used to evaluate the morphometric parameters of the plant, such as height and biomass [51]. The digital biovolume was calculated from the three orthogonal images of the same plant according to the formula [52]:

$$\sum pixelsideview0{}^{\circ}+\sum pixelsideview90{}^{\circ}+\log\left(\sum \frac{pixeltopview}{3}\right)$$

The color classes that were chosen were determined experimentally for each experiment by examining the hue histogram. Here, only yellow and dark green are shown. The number of fruits was recorded in full maturity from the second truss. In addition, the seed number for each fruit from this truss was also determined. 

### 2.6. Expression Constructs

The expression constructs of *HSBP1* wild-type and *HSBPm* genes were cloned either with an N-terminal green fluorescence protein (GFP) or HA-tag, using the appropriate primers (Table S1). Plasmids encoding for HsfA1a, HsfA2, and HsfB1 as well as *PGmHsp17-CI*::GUS have been described elsewhere [20,53]. For the repressor assay, the pRT103 GUS vector was used with three inserted heat stress element (HSE) oligonucleotides downstream of the TATA box of the CaMV35S promoter. Therefore, the high constitutive activity of the CaMV35S of the vector was reduced in the presence of HSE-binding factors [53].

### 2.7. Protoplast Preparation and GUS Reporter Assays

Mesophyll protoplasts from sterile grown tomato plants cv. *Moneymaker* were isolated and transformed as previously described [22]. Fifty thousand protoplasts were transformed with a total of 10 μg of plasmid DNA per sample consisting of 0.5 μg of each Hsf or HSBP1-expressing plasmid and 1 μg of the reporter plasmid DNA construct. The total amount of plasmids was complemented to 10 μg with a pRT-Neo mock plasmid. Following transformation, protoplasts were incubated for 6 hours at 25 °C, and β-glucuronidase (GUS) activities were determined as described previously [53,54]. Alternatively, protoplasts were exposed to the indicated temperature in a water bath, collected by centrifugation, and then snap frozen in liquid nitrogen.

### 2.8. RNA Extraction and Transcript Analysis

Total RNA was extracted using the E.Z.N.A. Plant RNA Kit (Omega Bio-Tek, Norcross, GA, USA) following the manufacturer’s instructions. cDNA was synthesized using 1 μg of total RNA with Revert Aid reverse transcriptase (Thermo Scientific) following the manufacturer’s protocol. The expression of *HSP70-1*, *Hsp70-5*, and *HsfA2* genes was determined using quantitative real-time PCR (qRT-PCR) on a StepOnePlus (Thermo Fisher Scientific). The reaction (10 μl) consisted of gene primers (Table S1), PerfeCTa^®^ SYBR^®^ Green FastMix Low ROX^™^ (Quanta Biosciencies), and the template. Thermal cycling conditions were 95 °C/3 min followed by 95 °C/15 s, 60 °C/30 s, and 72 °C/30 s for 40 cycles. Gene primers were designed using PRIMER3 (www-genome.wi.mit.edu/cgi-bin/primer/primer3.cgi/). Data were analyzed by standard methods [55] and presented as relative levels of gene expression using the EF1α (Solyc06g005060) and Actin (Solyc11g005330) genes as internal standards.

### 2.9. Microscopy

The subcellular localization of GFP-tagged HSBP1 and HSBP1m proteins was performed under a Leica SP5 confocal laser-scanning microscope. GFP was excited at 488 nm and mCherry at 561 nm. ENP1-mCherry was used as a nuclear marker protein [56]. Fluorescence emission was measured at 490–548 nm (GFP) and 570–656 nm (mCherry). 

### 2.10. Orthology Search and in Silico Structure Prediction

Orthologous genes to *At-HSBP1* were identified via OrthoDB [57]. The secondary structure was predicted and visualized by I-TASSER based on the models with lower C scores [58]. CCBuilder with an implemented BUDE algorithm was used for the calculation of force fields scores for interaction energies based on an analysis of the wild-type MSESIISKIDEMGNRIDELE or mutant MSESIISKIDEIGNRIDELE peptide, using the following parameters: trimer oligomeric state, radius 5.1, Pitch 226, and interface angle 24 [59]. 

### 2.11. Transcriptome Data

Information on the expression of selected genes was obtained by the TOMEXPRESS database. The comparison of expression profiles between Sl-HSBP1 and Hsfs or Hsps was done by Pearson correlation analysis based on transcript levels across 106 individual samples of different organs, tissues, and tomato genotypes at different developmental stages. 

## 3. Results

### 3.1. Expression of Putative HSBP Genes in Tomato

**Figure 1:**
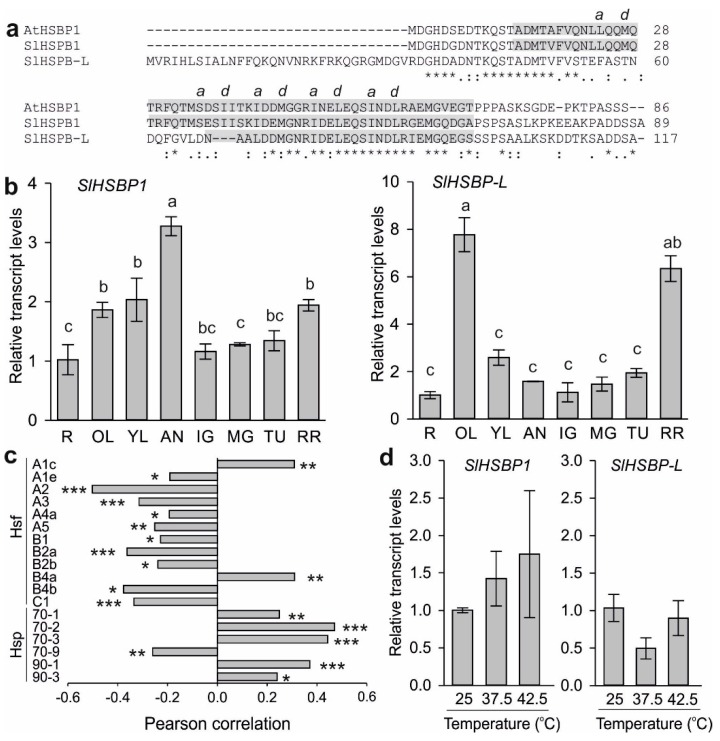
Domain structure and expression analysis of tomato heat shock binding protein (HSBP) genes. (**a**) Amino acid sequence alignment of *A. thaliana* and tomato HSBP proteins by Clustal W. The coiled-coil region is depicted in grey background. Hydrophobic residues at the a and d positions are shown on top. (**b**) Transcript levels of tomato *SlHSBP1* and *SlHSBP-L* in different organs. R: root; OL: old leaf; YL: young leaf; AN: anther; IG: immature green fruit; MG: mature green fruit; TU: turning fruit; RR: red ripe fruit. Analysis was done by qRT-PCR using EF1a as a housekeeping gene, and the transcript levels were normalized to the root sample. Values are the average of three replicates, and error bars indicate the standard deviation. Different letters denote significant differences (*p* < 0.05) based on ANOVA analysis and Duncan’s multiple range test. (**c**) Pearson correlation analysis of transcript levels of *SlHSBP1* with heat shock transcription factors (Hsfs) and heat shock proteins (Hsps) in tomato organs and tissues. Transcript levels were obtained from the TOMEXPRESS database (*n* = 106). Asterisks indicate significance (* *p* < 0.05, ** *p* < 0.01 *** *p* < 0.001) (**d**) Transcript levels of *SlHSBP1* and *SlHSBP-L* in young tomato leaves exposed to different temperatures for 1 hour. Values for panels b and d are the average of three biological replicates ± SD.

Two genes, *Solyc01g067905* and *Solyc12g099570*, are annotated as orthologs to *A. thaliana HSBP1* [57]. Solyc01g067905 encodes for a 12.9-kDa and Solyc12g099570 encodes for a 9.8-kDa protein. Solyc12g099570 shares a higher sequence similarity to At-HSBP1 than Solyc01g067905, and it likely shares similar conformation, since the *a* and *d* hydrophobic residues in five hydrophobic heptad repeats are conserved (Figure 1a). The coiled-region of Solyc12g099570 is of similar length to that of At-HSBP1, while this domain of Solyc01g067905 shows variations in length and composition (Figure 1a). Moreover, Solyc01g067905 has an extended N-terminal region compared to other Heat shock binding proteins (HSBPs) (Figure S1). Based on this, Solyc12g099570 is annotated as HSBP1, and Solyc01g067905 is annotated as the HSBP-like (HSBP-L) protein. 

The transcript levels of both genes under non-stress conditions in different tomato tissues were determined by qPCR. *SlHSBP1* shows the highest levels in anthers, and slightly increased levels in leaves compared to roots (Figure 1b). *SlHSBP-L* levels peak in old leaves and are increased in red ripe fruits (Figure 1b). As HSBPs are considered negative regulators of Hsfs and consequently of the transcription of HS-induced genes, we examined the correlation of *SlHSBP1* with that of all tomato Hsfs and cytosolic Hsp101, Hsp90, Hsp70, and small Hsps, as several of these genes are differentially regulated in various tissues and developmental stages [9]. Pearson correlation was performed on the basis of publicly available RNAseq data for *SlHSBP1* and *HsfA2*, but not for *SlHSBP-L*, for which transcriptome data is not currently available [60]. Indeed, a weak but significant negative correlation calculated across 106 samples was detected for *SlHSBP1* with 10 Hsfs and *Hsp70-9* (Figure 1c). As many of these genes are regulated in an Hsf-dependent manner, the negative correlation is in agreement with the assumed repressor function of *SlHSBP1*. Remarkably, *HsfA1c* and *HsfB4a* as well as *Hsp70-1*, *Hsp70-2*, *Hsp70-3*, *Hsp90-1*, and *Hsp90-3* showed a weak but significant positive correlation (Figure 1c). These results suggest that *SlHSBP1* and the above-mentioned genes are regulated by similar, probably Hsf-independent, transcriptional networks. 

As HSBPs have been described as HS-induced, the levels of HSBP transcripts were determined in leaves exposed for 1 hour at 37.5°C or 42.5 °C, or kept for the same time at 25 °C as control (Figure 1d). Remarkably, neither *SlHSBP1* nor *SlHSBP-L* showed a significant change in transcript levels after one hour at high temperatures.

### 3.2. A Mutation of the Zipper Affects the HSBP Functionality 

The homology of *SlHSBP1* to *AtHSBP1* and the correlation of its expression profile with genes coding for central Hsfs marked *SlHSBP1* as a promising candidate for the manipulation of the HSR in tomato. A TILLING approach based on EMS mutagenesis of the cv. Red Setter was carried out [46], and a population of 5200 M3 families was screened with SlHSBP1 specific primers, leading to the identification of a single-base substitution G761A. The mutation causes a change of Met to Ile at position 45 (M45I). Using the CCBuilder, BUDE force fields scores for interaction energies between 20 amino acid residue peptides containing the heptad repeat with the mutation, as well as the N-terminal and C-terminal repeats in the coiled-coil region; values of −22.4 kJ/mol for SlHSBP1 (translating to K_D_ = 100µM at 20 °C) and −29.4 kJ/mol for mutated SlHSBP1 (named SlHSBP1m) (translating to K_D_ = 6µM) were predicted. These results suggest a potential further stabilization of the oligomeric complex due to the Met to Ile substitution.

**Figure 2:**
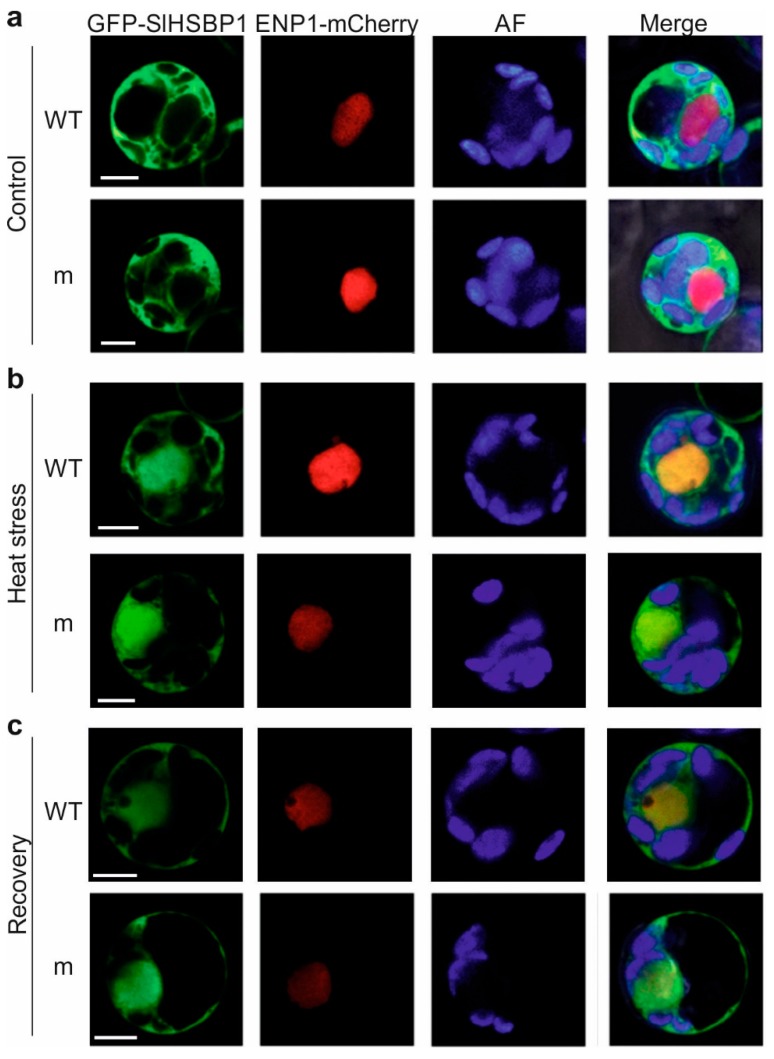
Subcellular localization of GFP-tagged HSBP1 (WT) and HSBP1m (m) proteins (**a**) before (control) and (**b**) after heat stress, or (**c**) during the recovery phase. Protoplasts were either kept at 25 °C or exposed to 40 °C for 1 hour (heat stress), and then allowed to recover for 1.5 hours at 25 °C. AF: chlorophyll autofluorescence. ENP1-mCherry is a nuclear marker protein. Scale bars are 5 μm.

The localization of both the wild-type and mutated SlHSBP1 protein, which was N-terminally tagged with GFP, was examined in control (non-stressed) and HS protoplasts (Figure 2). Under non-stress conditions, both proteins were localized in the cytosol (Figure 2a), while after 1 hour of HS (Figure 2b) and during the following recovery (Figure 2c), SlHSBP1 and mutated SlHSBP1 (HSBP1m) showed nucleocytoplasmic distribution. These results are in agreement with the nuclear translocation of AtHSBP1 in stressed cells [41], and demonstrate that the translocation is not affected by the Met to Ile mutation (Figure 2).

**Figure 3:**
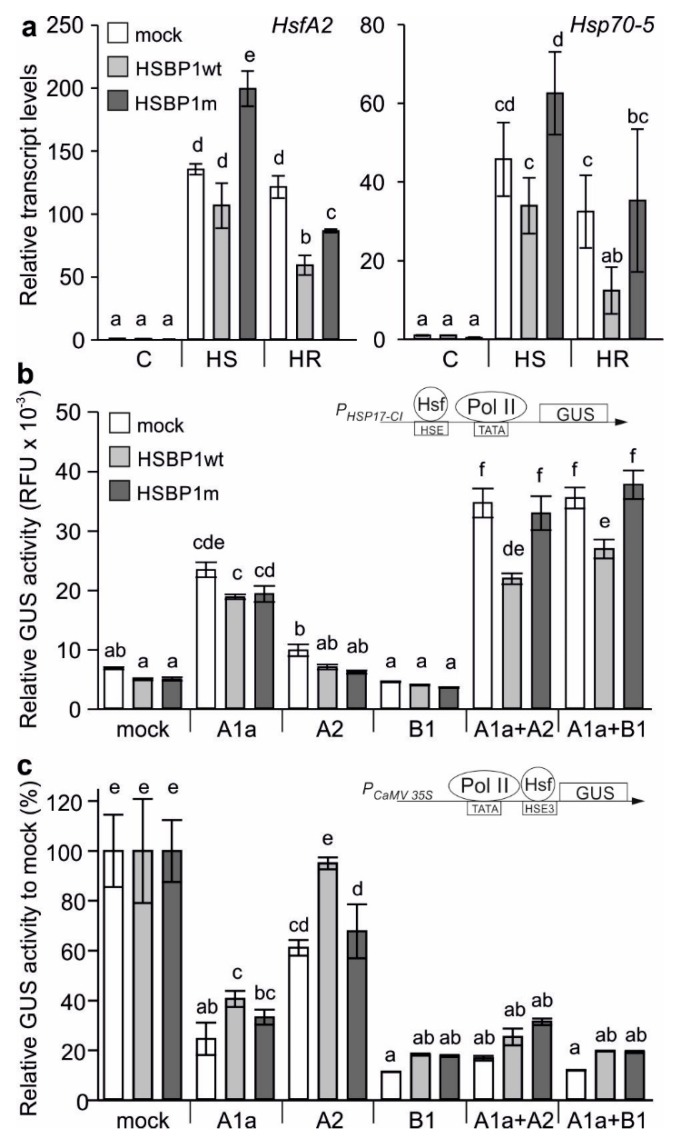
Effect of SlHSBP1 and SlHSBP1m on properties of major Hsfs. (**a**) Transcript levels of *HsfA2* and *Hsp70-5* in protoplasts expressing *SlHSBP1* or *SlHSBP1m* in comparison to mock samples. Protoplasts were either kept at 25 °C (control) or exposed to 40 °C for 1 hour (HS) and then allowed to recover for 1.5 hours at 25 °C (HR). Values are expressed relative to the mock control sample and are the average of three replicates and error bars are ±SE. EF1a was used for normalization. (**b**) The transactivation activity of single or combinations of HsfA1a, HsfA2, or HsfB1 in the presence of HSBP1 or HSBP1m. P_HSP17-CI_::GUS was used as reporter. (**c**) GUS repressor for elucidation of Hsf DNA binding capacity using the *P_CaMV 35S-HSE3_*::GUS reporter. The repression of activity is calculated relative to a sample where no Hsf was expressed for each *HSBP1*, *HSBP1m*, and mock control. Values are the average of three individual replicates ±SD. RFU: relative fluorescence unit. Different letters denote significant differences (*p* < 0.05) based on ANOVA analysis and Duncan’s multiple-range test.

We further inspected the effect of the mutation on the functionality of SlHSBP1. HA-tagged SlHSBP1 and HSBP1m were expressed in protoplasts, which were either kept at 25 °C (C), or exposed to 40 °C for 1 hour (HS) and then returned to 25 °C for another 1.5 hours (HR) (Figure 3a). Protoplasts expressing *SlHSBP1* accumulated levels of *HsfA2* and *Hsp70-5*, which was similar to the mock sample not overexpressing any HSBP protein after HS, but had significantly lower levels of both genes in the recovery samples (Figure 3a). Interestingly, *HsfA2* and *Hsp70-5* showed increased levels in stressed protoplasts expressing *SlHSBP1m* when compared to SlHSBP1-expressing cells or to mock stressed samples (Figure 3a). On the basis of these results, we can infer that SlHSBP1m has a stimulatory effect during HS, and is also less inhibitory under recovery than the wild-type protein.

HSBP1 is a negative regulator of Hsfs, as shown in other plants [41,43,44]. Therefore, we examined the effect of SlHSBP1 and SlHSBP1m on the activity of the two major tomato Hsfs, namely *HsfA1a* and *HsfA2*, using a GUS activity assay with the *P_Hsp17-CI_*::GUS reporter construct (Figure 3b). In parallel, we used HsfB1 as control since, on one hand—according to the current model—HSBP only regulates HsfA-type proteins [41,43]. However, on the other hand, HsfB1 is also a co-activator of HsfA1a [61]. Neither SlHSBP1 nor SlHSBP1m caused a significant reduction in the transactivation activity of HsfA1a and HsfA2, but also had neither had an effect on HsfB1. Interestingly, only SlHSBP1 had a negative effect on the activity of HsfA1a/HsfA2 and HsfA1a/HsfB1 complexes, while SlHSBP1m has no effect (Figure 3b).

A GUS repressor assay was used to further examine whether the observed reduced Hsf activity in the presence of SlHSBP1 is due to an altered Hsf DNA binding capacity (Figure 3c). Here, HSE3 has been cloned between the TATA box of the CaMV 35S promoter and the initiation codon of the GUS gene, and therefore, the binding of an Hsf to the HSE results in reduced GUS activity [53]. As previously established, HsfA1a, HsfA2, and HsfB1 bind to the used promoter (Figure 3c, white bar [36,38]). SlHSBP1, but not SlHSBP1m, reduced the DNA binding capacity of HsfA1a and HsfA2, and had no effect on HsfB1. Remarkably, both SlHSBP1 and SlHSBP1m caused a mild reduction in the DNA binding capacity of HsfA1a/HsfA2 and HsfA1a/HsfB1 complexes, which is consistent with the mild effect on the DNA binding of the individual Hsfs (Figure 3c). 

### 3.3. Thermotolerance of SlHSBP1 Plants

The experiments described in the previous sections provide evidence that the Met to Ile substitution, at position 45, has a significant effect on the functionality of SlHSBP1. Thus, we examined whether the mutation affects the capacity of plants to tolerate heat stress. To reduce the rate of background mutation in the tomato genome, the mutant line, which is homozygous for the *HSBP1* G761A mutation, was backcrossed to the wild-type Red Setter. The BC1F2 segregant plants were selected for homozygosity in the wild-type (*SlHSBP1wt*) and mutated (*SlHSBP1m*) allele. BC1F3 tomato plants carrying the *SlHSBP1wt* or *SlHSBP1m* allele were used for thermotolerance experiments.

**Figure 4:**
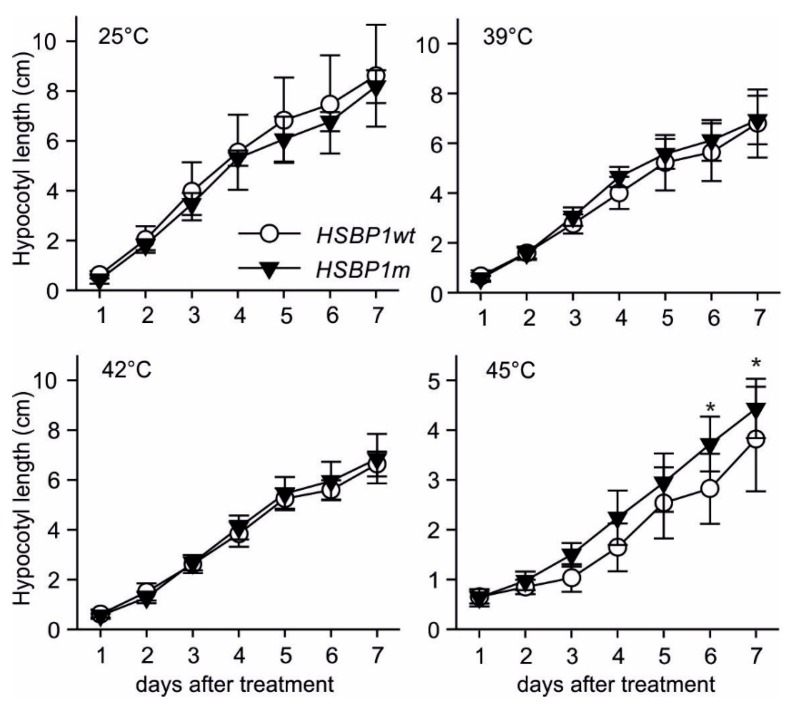
Effect of heat stress on hypocotyl elongation of *SlHSBP1wt* and *SlHSBP1m* seedlings. Four-day-old seedlings were exposed to the indicated temperatures for one hour, and then allowed to grow for 7 days at 25 °C in the dark. Thermotolerance is estimated by the ability of seedlings to recover from stress based on their hypocotyl growth. Values are an average of 10 seedlings ±SD. Asterisks indicate significant difference (*p* < 0.05) between the two genotypes for the indicated day based on Student’s *t*-test.

Four-day-old seedlings were exposed to different temperatures ranging from 25 to 45 °C for 1.5 hours; then, the hypocotyl elongation rate was determined for the following 7 days. *SlHSBP1wt* and *SlHSBP1m* seedlings showed a similar growth rate under non-stress or when exposed to 39 °C or 42 °C stress treatment (Figure 4). In turn, *SlHSBP1m* seedlings have an increased elongation rate after the 45 °C treatment when compared to *SlHSBP1wt* (Figure 4). Therefore, *SlHSBP1m* seedlings show higher tolerance to strong HS treatment.

Next, six-week-old plants were exposed to a 39 °C treatment for 1 hour for five consecutive days. Every day, various physiological parameters were recorded using the plant phenotyping platform. As controls, HsfA1a overexpression (A1OE) and co-suppression lines (A1CS) were included as well as their wild-type background cv. Moneymaker [22]. The experiment was figured out to verify whether the effect of the *SlHSBP1* mutation was similar to the effect of *HsfA1a* in overexpression lines. Comparisons were made on one side between *SlHSBP1wt* and *SlHSBP1m*, and on the other side between the Moneymaker and *HsfA1a* transgenic lines. 

**Figure 5:**
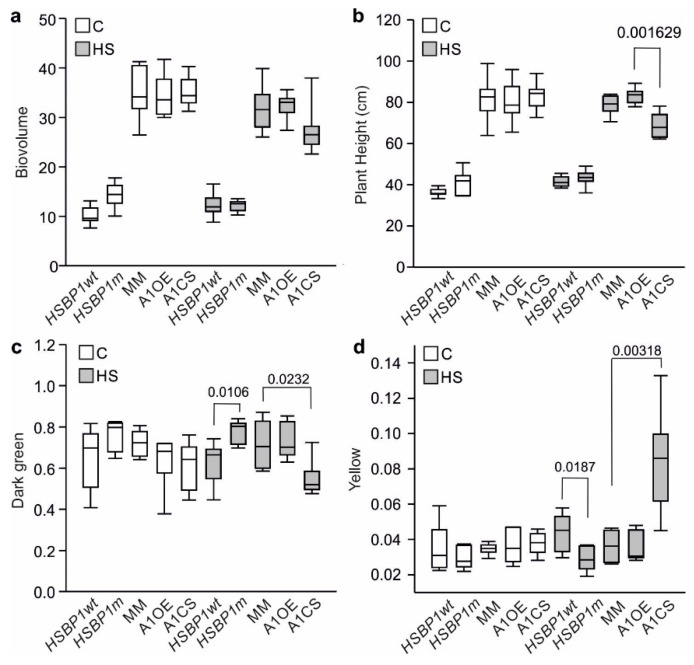
Physiological parameters of control and heat-stressed tomato plants. (**a**) Biovolume, (**b**) Plant height, (**c**) Dark green, and (**d**) Yellow color of *SlHSBP1wt*, *SlHSBP1m*, *Moneymaker* (MM), A1OE, and A1CS plants kept either under control conditions (25 °C; C) for 4 days or exposed to 1 hour of heat shock (HS) at 39 °C every day. Measurements were conducted by a LemnaTec Scanalyzer 3D System. Box plots show the distribution of measurements from eight plants for each parameter as well as the median and the upper and lower quartiles. Statistical differences (*p* < 0.05) based on pairwise comparisons by Student’s t-test of the mutant or transgenic plants with *SlHSBP1wt* or Moneymaker, respectively, are indicated on top of the plots.

Stressed and non-stressed *SlHSBP1wt* and *SlHSBP1m* plants did not show any significant differences in plant height or biovolume after 4 days, while only A1CS showed a significantly reduced height after 4 days of stress when compared to Moneymaker (Figure 5a,b). The dark green color is associated with healthy tissue, while yellow is an indicator of chlorosis [61]. No differences between the mutant and the transgenic lines were observed in non-stressed plants after 4 days of treatment (Figure 5c,d). Instead, *SlHSBP1m* shows a significantly higher dark green color and a lower yellow color compared to *SlHSBP1wt* (Figure 5c,d). A1OE has similar dark green and yellow values to Moneymaker, whilst A1CS shows a significantly reduced dark green and increased yellow color. These results indicate a higher thermotolerance for *SlHSBP1m* and enhanced thermosensitivity for A1CS. The latter gives strong indication that the thermotolerance in response to the applied stress treatment is Hsf-dependent.

**Figure 6:**
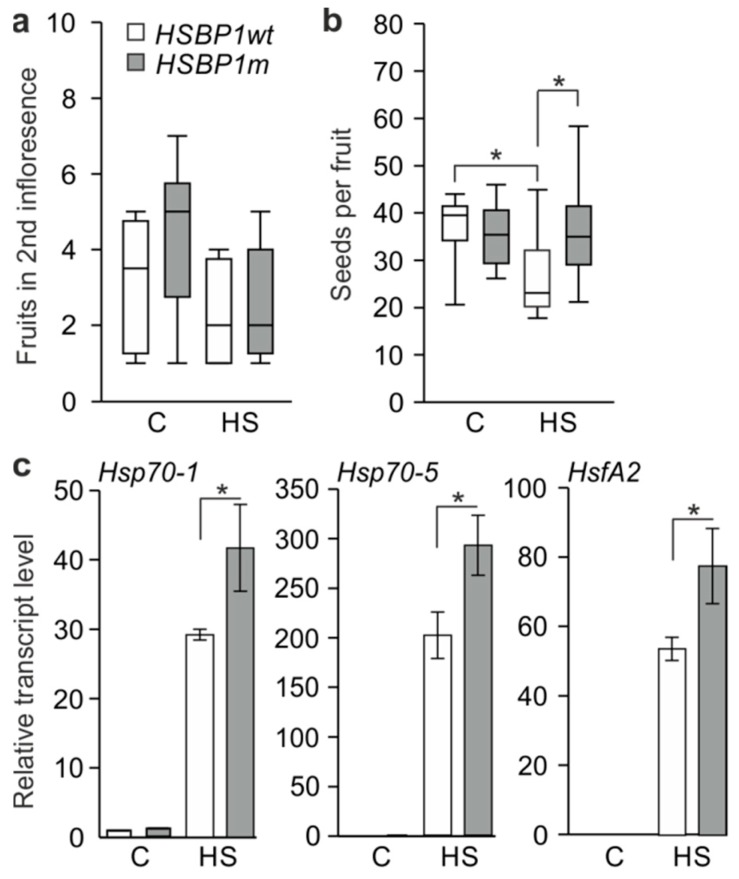
Thermotolerance of *SlHSBP1wt* and *SlHSBP1m* lines. (**a**) Number of fruits from the second trust and (**b**) number of seeds per fruit are shown in box plots, including the median and the upper and lower quartiles (*n* = 8) in control (non-stressed) and heat-stressed *SlHSBP1wt* and *SlHSBP1m* plants treated as described above. Asterisks indicate significant differences between the indicated samples, based on Student’s *t*-test (*p* < 0.05). (**c**) Transcript levels of *Hsp70-1*, *Hsp70-5*, and *HsfA2* in control and heat-stressed *SlHSBP1wt* and *SlHSBP1m* plants. Expression values are normalized against the *SlHSBP1wt* control sample determined by qRT-PCR. Values are the average of three biological replicates ±SD. Asterisks denote a significant difference (*p* < 0.05) based on pairwise t-test analysis.

Furthermore, after the treatment, the plants were allowed to grow under non-stress conditions, and the fruit set and seed number per fruit were recorded (Figure 6a,b). Although the fruit number was not affected by the treatment or by the mutation (Figure 6a), the number of seeds per fruit was significantly reduced in *SlHSBP1wt*-stressed plants but not in *SlHSBP1m* (Figure 6b). Overall, the *SlHSBP1m* plants exhibit increased thermotolerance compared to the *SlHSBP1wt*, which was probably due to the increased accumulation of Hsfs and Hsps.

The expression profiles of the stress-induced *HsfA2*, *Hsp70-1,* and *Hsp70-5* were monitored in *SlHSBP1wt* and *SlHSBP1m* control and stressed leaves (Figure 6c). In all cases, the levels of the transcripts are higher in *SlHSBP1m* stressed leaves when compared to *SlHSBP1wt*, which is in agreement with the results described earlier using protoplasts, while no significant differences were detected for non-stressed samples between the lines.

**Figure 7:**
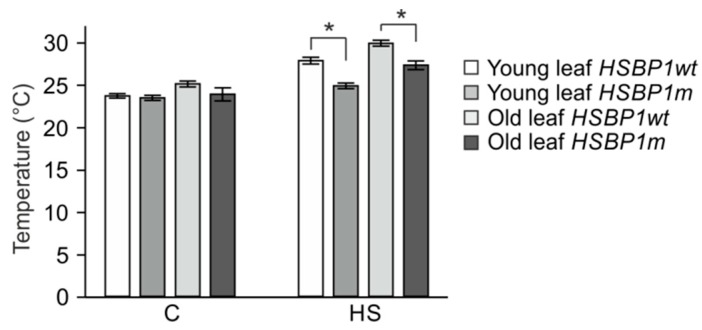
Effect of *SlHSBP1* mutation on leaf surface temperature. Young and old leaf temperature of *SlHSBP1wt* and *SlHSBP1m* plants after a gradual exposure to HS that reached 36 °C at maximum or were kept at 25 °C for the same period as the control (sample C). Values are the average of eight plants from a representative experiment. Asterisks denote significant differences (*p* < 0.05) based on t-test analysis.

Plants cool their canopies when exposed to high temperatures via transpiration; therefore, the maintenance of lower leaf temperature is a major protection mechanism against HS. Consequently, we monitored the leaf surface temperature of plants subjected to a gradual daily temperature increase that reached a maximum of 36 °C. Consistent with the higher thermotolerance observed with image analysis, heat-stressed *SlHSBP1m* young and old leaves were able to maintain a lower temperature when compared to *SlHSBP1wt* leaves (Figure 7).

## 4. Discussion

### 4.1. Characterization of Tomato HSBP1 Protein

HSBP is a well-studied protein in several eukaryotes with a conserved function as an Hsf repressor, and is therefore a potential target for manipulation and, subsequently potential breeding interest for crops. An orthology search revealed the presence of two putative orthologues of the *A. thaliana HSBP* gene in tomato (Figure 1; Figure S1), with *SlHSBP1* showing the highest sequence homology and predicted structure similarity (Figure 1). SlHSBP-L has an extended N-terminal region that is also found in other plant HSBP orthologues, including *Glycine max* and *Brassica rapa*, but also a variable N-terminal region of the helix region compared to SlHSBP1 (Figure 1 and Figure 2). Interestingly, neither *SlHSBP1* nor *SlHSBP-L* are heat stress-induced in leaves, which might indicate regulation at the post-transcriptional level or even induction in specific tissues and cell types (Figure 1). Under non-stress conditions, *SlHSBP-L* shows high transcription in old leaves and red ripe fruits, with both mature tissues progressing to senescence (Figure 1). However, at this stage, we cannot conclude on the possible functionality of *SlHSBP-L*, as it requires further studies. 

*SlHSBP1* shows enhanced transcription levels in reproductive tissue such as anthers, which is similar to the *HSBP* genes from other plants showing increased expression in siliques, embryos, and panicles [41,42,43,44]. We observed an accumulation of the GFP-tagged SlHSBP1 in the nucleus of protoplasts exposed to heat stress (Figure 2), which is consistent with the findings for AtHSBP1 [41]. Interestingly, in our recent study, the SlHSBP1 protein was identified in a proteomic analysis of leaves, and also showed no accumulation in response to heat stress, but was significantly reduced in both control and heat-stress leaves from transgenic HsfB1 knockdown plants [30]. Therefore, the regulation of *SlHSBP1* in tomato seems to be different than in other plants. 

It is of note that several *Hsfs* such as *HsfA2* were expressed in anthers and early stages of pollen development in tomato, which could support a regulatory role of SlHSBP1 for the developmental regulation of *Hsfs* [14,26,62]. In agreement with this, we found a significant negative correlation between *SlHSBP1* and several *Hsfs* in various tomato tissues and organs, supporting a potential negative regulatory function of SlHSBP1 for stress-induced but also developmentally regulated genes (Figure 1). Within this line, SlHSBP1 had a negative effect on DNA binding when HsfA1a and HsfA2 were expressed alone or in combination, while it did not affect HsfB1 function (Figure 3). These results are in agreement with previous studies where a repressor function of HSBP1 has been shown [41,43,44].

In contrast to this finding, several *Hsp70* and *Hsp90* genes—both stress-induced and constitutively expressed—showed a positive correlation in expression with SlHSBP1 (Figure 1). This indicates that SlHSBP1 belongs to the same regulatory module with Hsps, which might be Hsf-independent.

A Met-to-Ile exchange in the *d* position of a central heptad repeat is subtle, as both residues are hydrophobic and typically found in coiled-coil structures. Nevertheless, we observed an altered functionality for the SlHSBP1 mutant protein (SlHSBP1m). SlHSBP1m is mainly cytosolic, and accumulates under stress conditions in the nucleus as the wild-type protein (Figure 2). Therefore, the mutation does not affect the nucleocytoplasmic distribution of the protein. However, it has a weaker repressor effect on the transactivation activity of Hsf complexes (Figure 3). 

HSBP builds functional hexamers that consist of two trimers [39,40]. We found by prediction that the mutation might have a stabilizing effect on the trimer. Although more biophysical information is required, based on this, one could suggest that HSBP1 forms a heterotrimer with the coiled-coil domain of the HsfA family proteins. A higher affinity for self-oligomerization would inhibit the regulatory function, which in turn results in higher activity for the HsfAs. Indeed, protoplasts expressing SlHSBP1m accumulated higher levels of *HsfA2* and *Hsp70-5* upon HS when compared to mock samples or *SlHSBP1*-expressing cells (Figure 3). Based on these results, we conclude that the mutation causes a partial loss of SlHSBP1 function. 

### 4.2. SlHSBP1m Plants Exhibit Higher Thermotolerance

*SlHSBP1m* tomato plants show enhanced thermotolerance in response to a cycle of four days heat stress for 1 hour. Although the growth of the *SlHSBP1m* or *SlHSBP1wt* plants was not significantly affected during the treatment, the dark green color of the plants, which was indicative of the health of the tissue, was higher in *SlHSBP1m* plants, while the yellow color, which was indicative of reduced chlorophyll, was reduced when compared to *SlHSBP1wt* (Figure 5). These results are in agreement with the ability of *SlHSBP1m* leaves to maintain a lower leaf surface temperature, which was probably due to the enhanced transpiration rate, and can protect the photosynthetic apparatus from thermal damages (Figure 5). Furthermore, *SlHSBP1m* plants accumulated higher transcript levels of *Hsp70-1*, *Hsp70-5*, and *HsfA2* as typically heat stress-induced genes (Figure 6). Remarkably, *SlHSBP1m* seedlings exhibited higher thermotolerance after an acute 45 °C stress compared to *SlHSBP1wt* seedlings, suggesting that SlHSBP1 is involved in basal thermotolerance (Figure 4).

In addition, *SlHSBP1m* plants produced a number of fruits similar to *SlHSBP1wt* but with a higher number of seeds (Figure 6). Considering that developing flower buds also received the stress during the time of the treatment, we can assume that the mutation in *SlHSBP1* resulted in the enhanced protection of gametophytes. Altogether, these results point to a higher capacity of *SlHSBP1m* plants to withstand heat stress.

In conclusion, we were able to show that a tomato mutant genotype carrying a missense mutation in *SlHSBP1* gene exhibits increased thermotolerance. In contrast to maize, the mutation does not cause significant developmental defects; therefore, this mutant line could be used in breeding programs for the generation of heat stress-resilient plants. 

## Supplementary Materials

The following are available online at https://www.mdpi.com/2073-4425/10/7/516/s1, Figure S1: Secondary structure of tomato HSBP proteins. Table S1: Oligonucleotides used in this study.
