# Supplementary material for: Identification and Characterization of a Thermotolerant TILLING Allele of Heat Shock Binding Protein 1 in Tomato
Source: Genes (Basel). 2019 Jul 7;10(7):516. doi: 10.3390/genes10070516 (PMC6678839; doi:10.3390/genes10070516)
Supplement: Supplementary file 1 [file genes-10-00516-s001.pdf]

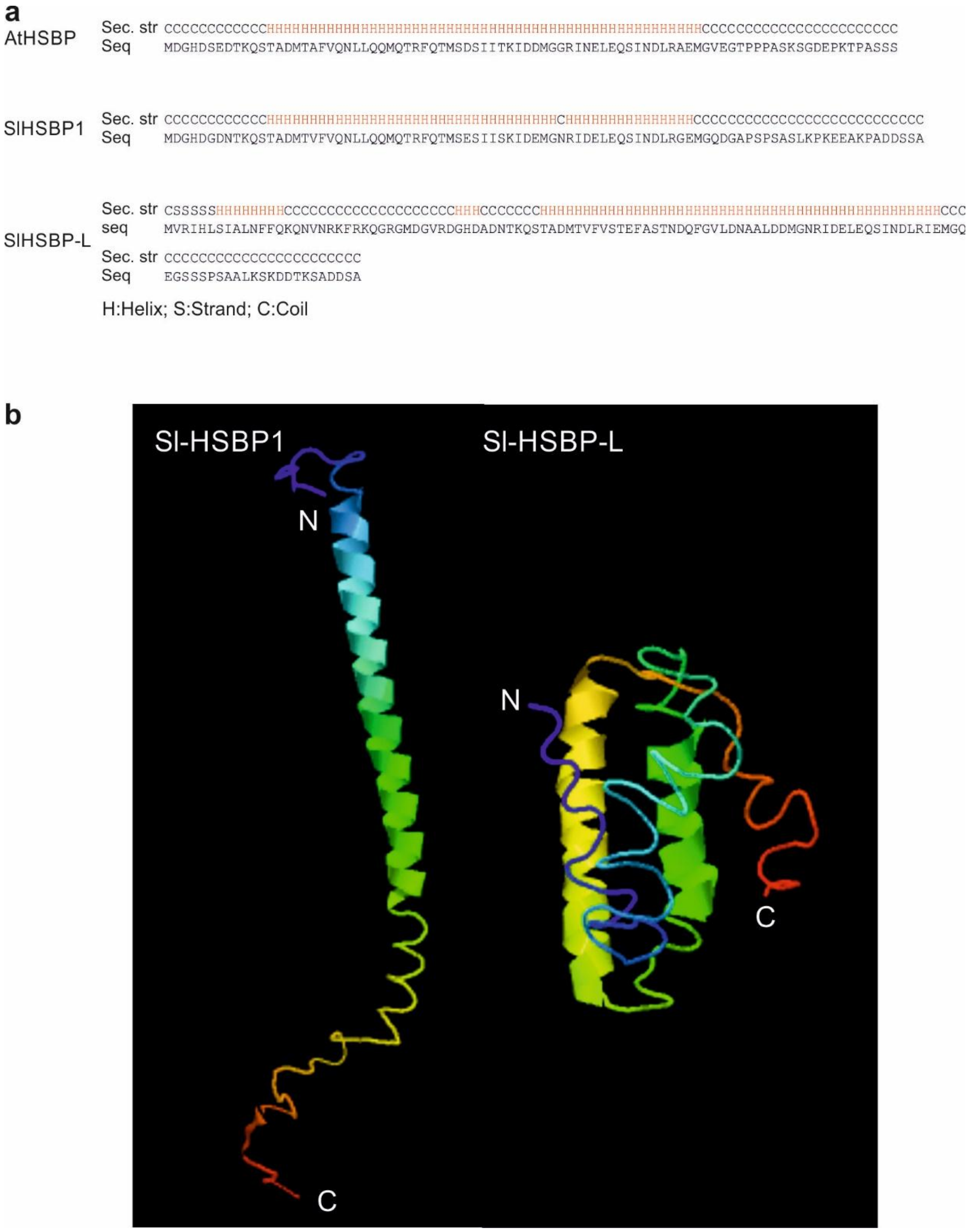

Figure S1. Secondary structure of tomato HSBP proteins. (**a**) Sequence and predicted secondary structure of *A. thaliana* and tomato HSBP proteins. C: Coil, H: Helix and S: strand. (**b**) Secondary structure was predicted and visualized by I-TASSER.

Table S1. Oligonucleotides used in this study

Gene (accession ID)	Name	Sequence (5′ > 3′)	Amplicon length (bp)
EF1a (Solyc06g005060)	EF1a-F	GGAACTTGAGAAGGAGCCT	158 bp
	EF1a-R	CAACACCCACAGCAACAGT	
Actin (Solyc11g005330)	Actin-F	GCTGACCGTATGAGCAAGG	72 bp
	Actin-R	CGGTGGAGCAACAACCTTA	
HSBP1 (Solyc12g09957[illegible]	SlHSBP1-F	GACAATGTCCGAATCAATC	161 bp
	SlHSBP1-R	TGCACTAGAATCATCTGCTG	
HSBP-L (Solyc01g06790[illegible]	SlHSBP-L-	TGGAGTCCTTGACAATGCTG	161 bp
	SlHSBP-L-	CAGAATCATCAGCAGATTTT	
Hsp70-1 (Solyc06g07602[illegible]	Hsp70-1-F	GCTCGAGGGCATCTGTAATC	164 bp
	Hsp70-1-R	GAAACCAACTAGTATCTTTA	
Hsp70-5 (Solyc04g01144[illegible]	Hsp70-5-F	GGAAGTGGACTAAGCTCCA	123 bp
	Hsp70-5-R	CGAAGGATATTTCTACATAC	
HsfA2 (Solyc08g062960)	HsfA2-F	AGGTGGATCCTGACAGATG	128 bp
	HsfA2-R	GCAAGCACCAGATCCTTGTT	
